# A Novel Pathogenic Frameshift Variant Associated With Holt–Oram Syndrome: A Case Report

**DOI:** 10.1155/crig/8875290

**Published:** 2026-09-16

**Authors:** Soyi Sarkar, Akshay N. Rana, Garrett Gianneschi, Peyman Bizargity

**Affiliations:** ^1^ Rutgers-New Jersey Medical School, Newark, New Jersey, USA, rutgers.edu; ^2^ Department of Neurology, Rutgers-New Jersey Medical School, Newark, New Jersey, USA, rutgers.edu; ^3^ Department of Genetics, Rutgers-New Jersey Medical School, Newark, New Jersey, USA, rutgers.edu

**Keywords:** congenital heart disease (CHD), Holt–Oram syndrome, sinus bradycardia, *TBX5*, upper limb defects

## Abstract

Holt–Oram syndrome is an autosomal dominant disorder characterized by congenital heart defects and distal upper‐limb abnormalities. Pathogenic variants in *TBX5*, located on chromosome 12q24.1, cause Holt–Oram syndrome. We report a 10‐year‐old boy with bilateral thumb anomalies, a large secundum atrial septal defect, and sinus bradycardia who was found to carry a maternally inherited *TBX5* frameshift variant, c.130del; p.(Ala44ProfsTer22). This early truncating variant is predicted to cause loss of function through protein truncation or nonsense‐mediated decay. To the best of our knowledge, this specific variant has not previously been described in the peer‐reviewed literature. Detailed electrocardiographic and ambulatory rhythm findings further characterize the conduction phenotype associated with this variant.

## 1. Literature Review

Holt–Oram syndrome (HOS) is a rare autosomal dominant disorder caused by pathogenic variants in *TBX5*, which encodes the T‐box transcription factor 5. The incidence of HOS is approximately 1 in 100,000 live births [1]. Pathogenic *TBX5* variants show complete penetrance for anterior upper‐limb abnormalities, whereas congenital heart disease (CHD) occurs in approximately 75%–91% of affected individuals [2]. Phenotypic expression is highly variable, and both clinical and molecular findings contribute to diagnosis.

The TBX5 transcription factor governs multiple developmental processes during embryogenesis. During limb development, *TBX5* expression is restricted to the forelimbs rather than the hindlimbs, which explains why pathogenic variants in *TBX5* cause upper‐limb malformations in HOS [2]. These abnormalities involve the thenar, carpal, and/or radial structures and may be unilateral, bilaterally symmetric, or bilaterally asymmetric [3]. Their severity ranges from hypoplastic, absent, or triphalangeal thumbs to severe radial‐ray reduction defects and phocomelia [4].

The TBX5 transcription factor also regulates cardiomyocyte proliferation and differentiation, cardiac septation, and development of the cardiac conduction system [2]. Cardiac malformations in HOS most commonly involve the atrial or ventricular septum, with atrial septal defects (ASDs) and ventricular septal defects (VSDs) being the most frequent lesions [5]. More complex congenital heart defects, including tetralogy of Fallot and left ventricular hypoplasia, have also been reported [2]. Regardless of whether a structural cardiac defect is present, affected individuals remain at risk for conduction disease, particularly sinus bradycardia and atrioventricular or bundle‐branch conduction abnormalities [1]. Variant type and location within *TBX5* may influence the expression of both cardiac and upper‐limb phenotypes [2].

This genotype‐phenotype variability was examined in a 2024 systematic review by Møller Nielsen et al. [6]. The authors identified 277 patients with HOS and categorized their variants as protein‐altering variants (missense variants and in‐frame duplications or deletions), protein‐truncating variants (nonsense variants, frameshift variants, or whole‐gene deletions), or other variants (including splice variants and exon‐level deletions or duplications). Among 108 unique *TBX5* variants, 43 (40%) were missense variants and 41 (38%) were protein‐truncating variants. Upper‐limb abnormalities and less severe CHD were more frequent in patients with protein‐truncating variants. Although arrhythmias were reported more often with missense variants overall, patients with arrhythmias and protein‐truncating variants were particularly likely to have bradyarrhythmias [6].

Although these genotype‐phenotype correlations provide insight into the clinical variability of HOS, they do not reliably predict the hemodynamic course of an individual cardiac lesion or the progression of conduction disease. Personalized assessment and longitudinal follow‐up are therefore essential [1]. Optimal management generally involves coordinated care among cardiology, orthopedics, and medical genetics.

In this case report, we describe a previously unreported early frameshift variant in *TBX5* that introduces a premature termination codon and is predicted to result in protein truncation or nonsense‐mediated decay. Reporting this protein‐truncating variant together with the proband’s detailed limb, structural cardiac, and conduction phenotypes expands the molecular spectrum of HOS and may inform the interpretation and surveillance of future patients carrying the same variant.

## 2. Case Presentation

The patient was a 10‐year‐old boy referred for evaluation of congenital upper‐limb anomalies and CHD. He was born at 37 weeks and 3 days of gestation by cesarean delivery because of maternal cardiomyopathy and preeclampsia. Fetal echocardiography demonstrated a large atrial communication, and hand anomalies were noted during the third trimester. At birth, both thumbs were dysplastic and abnormally positioned.

Postnatal echocardiography confirmed a large secundum ASD and two small muscular VSDs. The VSDs closed spontaneously, whereas the secundum ASD remained large and did not decrease in size during serial follow‐up. Because the ASD remained hemodynamically significant, the patient underwent pericardial patch closure through a right axillary approach on March 12, 2020, at 5 years of age. The operation and postoperative course were uncomplicated. Subsequent echocardiograms demonstrated no residual ASD, normal right‐heart dimensions, and normal biventricular systolic function.

Subsequent cardiac surveillance identified mild prolapse of both mitral valve leaflets with mild mitral regurgitation and mild ascending aortic dilatation, all of which remained stable on serial echocardiograms. Mild right ventricular compression from the pectus deformity was also observed, without evidence of right ventricular inflow or outflow tract obstruction.

A Holter monitor obtained at 7 years of age showed predominantly sinus rhythm with sinus arrhythmia, approximately 14 h of sinus bradycardia according to age‐specific reference ranges, and periods of shifting atrial pacemaker with a shorter PR interval. The minimum heart rate was 36 beats per minute during sleep, the average heart rate was 67 beats per minute, and the maximum heart rate was 185 beats per minute. The longest bradycardic episode lasted approximately 30 min. No significant pauses were identified; the longest R‐R interval was 1.7 s and occurred during sinus arrhythmia at the minimum heart rate. Ectopy was minimal, consisting of one ventricular ectopic beat and 10 supraventricular ectopic beats, and no symptoms were reported.

At 9 years of age, the patient experienced chest and abdominal discomfort at school and was found to have a heart rate in the high 40 s. He remained hemodynamically stable without syncope or altered responsiveness. An emergency‐department 12‐lead electrocardiogram demonstrated sinus bradycardia at 49 beats per minute with one junctional beat. A subsequent 3‐day Zio monitor showed a minimum heart rate of 39 beats per minute, a maximum heart rate of 197 beats per minute, and an average heart rate of 76 beats per minute. The predominant rhythm was sinus rhythm, with occasional junctional rhythm at low resting heart rates and rare premature atrial and ventricular contractions. No tachyarrhythmias or patient‐triggered events were recorded.

At cardiology follow‐up, a 12‐lead electrocardiogram showed predominantly normal sinus rhythm at 64 beats per minute with one junctional beat, a normal axis, a PR interval of 160 ms, a QRS duration of 92 ms, and a corrected QT interval of 419 ms. Right atrial enlargement was noted, but there was no ventricular pre‐excitation, ventricular hypertrophy, ectopy, or ST‐segment abnormality. The occasional junctional rhythm at low resting rates converted to sinus rhythm at higher rates. The patient remained active in soccer and basketball without syncope, exercise intolerance, or other exertional cardiac symptoms.

Past medical and surgical history included right inguinal hernia and hydrocele repair at 8 years of age, followed by contralateral inguinal hernia and hydrocele repair. In infancy, he had torticollis with positional plagiocephaly that was managed with helmet therapy.

Developmental history was notable for mildly delayed gross motor milestones, with independent walking at 15–16 months, and a period of speech regression at approximately 15–16 months that improved with speech therapy. He was later diagnosed with attention‐deficit/hyperactivity disorder; methylphenidate was discontinued because of mood‐related adverse effects. At the time of evaluation, he attended general education classes with age‐appropriate cognitive functioning, received occupational therapy for upper‐extremity range‐of‐motion limitations, and performed activities of daily living independently.

Family history was notable for the patient’s mother, who had a clinical diagnosis of HOS and more pronounced upper‐extremity malformations than the proband. She had no known intracardiac structural defect but was followed for cardiomyopathy that was considered likely related to chronic hypertension. Whole‐exome sequencing later confirmed that she carried the same pathogenic *TBX5* variant as the proband. The maternal grandmother also had a clinical diagnosis of HOS with subtle bilateral upper‐limb abnormalities and bradycardia requiring pacemaker placement at approximately 60 years of age. Neither the mother nor the maternal grandmother had intellectual disability. The family’s ancestry included Portuguese, Polish, Irish, and Italian descent.

**FIGURE 1:**
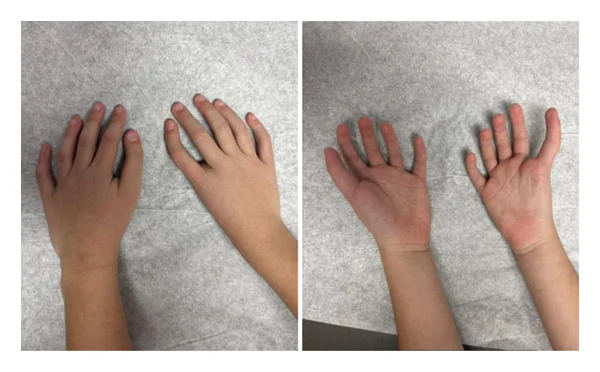
Laterally displaced triphalangeal right thumb and elongated, laterally displaced left thumb.

On physical examination, growth parameters were within the normal range. He had posteriorly rotated ears, pectus excavatum, a mild diastolic heart murmur, a right triphalangeal thumb, an elongated and laterally displaced left thumb (Figure 1), and bilateral pes planus. A 1‐cm hypopigmented macule was present on the back. The palate and toes were normal, and no joint hypermobility, abnormal skin elasticity, or impaired wound healing was identified.

Genetic testing performed in infancy was reportedly negative, although the original report was unavailable. Whole‐exome sequencing performed at 10 years of age identified a heterozygous maternally inherited pathogenic frameshift variant in *TBX5* (c.130del; p.(Ala44ProfsTer22)) in exon 2, confirming HOS. No pathogenic variants or variants of uncertain significance were identified in *FBN1* or in other analyzed genes associated with hereditary aortopathy or connective‐tissue disorders. The patient did not meet clinical diagnostic criteria for Marfan syndrome.

The patient continued longitudinal follow‐up with pediatric cardiology, including periodic echocardiography, annual electrocardiography, and ambulatory rhythm monitoring as clinically indicated.

## 3. Discussion

We report a 10‐year‐old boy with HOS characterized by bilateral thumb abnormalities, a large secundum ASD, two spontaneously closed muscular VSDs, and asymptomatic sinus bradycardia with occasional junctional rhythm. Whole‐exome sequencing identified a novel maternally inherited frameshift variant in *TBX5*, predicted to result in protein truncation or nonsense‐mediated decay, a recognized loss‐of‐function mechanism for HOS.

The *TBX5* gene, located on chromosome 12q24.1, encodes the TBX5 transcription factor, which is critical for cardiac and upper‐limb development. More than 90 pathogenic variants have been described, many of which are frameshift or nonsense variants affecting the DNA‐binding T‐box domain. Although c.130del; p.(Ala44ProfsTer22) is now listed in ClinVar as pathogenic, it had not been described in the peer‐reviewed literature or in large population databases such as gnomAD at the time of our evaluation.

Clinically, the proband exhibited bilateral anomalous thumbs, including a triphalangeal right thumb and an elongated, laterally displaced left thumb. These findings fall within the established upper‐limb spectrum of HOS, which ranges from subtle thenar or thumb abnormalities to radial aplasia and phocomelia. The mother had a more pronounced upper‐limb phenotype and cardiomyopathy without a known intracardiac structural defect, whereas the maternal grandmother had subtle limb abnormalities and later required a pacemaker for bradycardia. This three‐generation pattern illustrates substantial intrafamilial variable expressivity associated with the same *TBX5* variant.

The proband’s conduction phenotype was characterized by asymptomatic sinus bradycardia, sinus arrhythmia, shifting atrial pacemaker, and occasional junctional rhythm at low resting heart rates. Ambulatory monitoring documented minimum heart rates of 36–39 beats per minute, but no significant pauses, high‐grade atrioventricular block, sustained tachyarrhythmia, or clinically important ectopy. A follow‐up electrocardiogram showed a PR interval of 160 ms, a QRS duration of 92 ms, and a QTc of 419 ms. The appropriate conversion to sinus rhythm at higher heart rates and the absence of syncope or exercise intolerance argue against advanced sinus‐node dysfunction at the time of evaluation. Nevertheless, ongoing surveillance is appropriate because progressive conduction disease is a recognized feature of HOS, and the maternal grandmother’s pacemaker history supports a familial predisposition.

Interpretation of the bradycardia must also account for the proband’s structural and surgical cardiac history. The large secundum ASD remained unchanged during childhood and required surgical patch closure. Secundum ASDs may be associated with sinus‐node and atrioventricular‐node electrophysiologic abnormalities related to right‐sided volume loading, and sinus‐node function may also be affected by atrial surgery [7]. Although a large atrial defect could alter atrial anatomy, the available echocardiographic and operative records did not document displacement or injury of the sinoatrial node. The absence of a residual shunt and normalization of right‐heart size after repair are reassuring, but the relative contributions of intrinsic *TBX5*‐associated conduction susceptibility, prior atrial remodeling, and surgical repair cannot be definitively separated in this patient.

The patient also had pectus excavatum, bilateral inguinal hernias, bilateral pes planus, mild mitral valve prolapse, and mild ascending aortic dilatation. Pectus excavatum has occasionally been reported in HOS, whereas inguinal hernias and pes planus are not typical features and may be coincidental or represent broader phenotypic variability [2, 8]. Because the pectus deformity, mitral valve findings, and aortic dilatation raised concern for a coexisting connective‐tissue disorder, the exome analysis was reviewed for *FBN1* and other aortopathy or connective‐tissue genes. No pathogenic variants or variants of uncertain significance were identified, and the patient did not meet clinical criteria for Marfan syndrome. Continued cardiovascular surveillance remains appropriate given the mild ascending aortic dilatation.

In summary, this case expands the molecular spectrum of HOS by describing a novel early truncating *TBX5* variant in a child with classical upper‐limb and structural cardiac manifestations and a well‐characterized, currently mild conduction phenotype. It reinforces the marked intrafamilial variability of HOS and the importance of longitudinal rhythm surveillance. It also illustrates the need to consider structural heart disease and prior atrial surgery when attributing bradyarrhythmia solely to genotype.

## Funding

No funding was received for this manuscript.

## Consent

Written informed consent was obtained from the patient’s parent for publication of this case report, including any relevant clinical details and accompanying images. The parent was informed that all identifying information would be removed to preserve anonymity and that the publication may be accessible in print and electronic form. A copy of the signed consent form is available for review by the journal’s editorial office upon request.

## Conflicts of Interest

The authors declare no conflicts of interest.

## Data Availability

The data that support the findings of this study are available on request from the corresponding author. The data are not publicly available due to privacy or ethical restrictions.
